# Rational use of blood calcium determinations

**DOI:** 10.1590/1516-3180.2014.1324731

**Published:** 2014-05-28

**Authors:** Mario Ferreira-, Arnaldo Lichtenstein, Maria Mirtes Sales, Leandro Utino Taniguchi, Francisco José Bueno de Aguiar, Luiz Augusto Marcondes Fonseca, Nairo Massakazu Sumita, Alberto José da Silva Duarte

**Affiliations:** I MD, PhD. Attending Physician, Department of Internal Medicine, General Practice and Propaedeutics Service, Hospital das Clínicas, Faculdade de Medicina, Universidade de São Paulo, São Paulo, Brazil; II MD, PhD. Clinical Pathologist, Head of Flow Cytometry Laboratory, Central Laboratory Division, Hospital das Clínicas, Faculdade de Medicina, Universidade de São Paulo, São Paulo, Brazil; III MD, PhD. Intensive Care Physician, Discipline of Emergency Medicine, Hospital das Clínicas, Faculdade de Medicina, Universidade de São Paulo, and Hospital Sírio Libanês, São Paulo, Brazil; IV MD. Attending Physician and Supervisor of the Clinical Emergencies Division, Hospital das Clínicas, Faculdade de Medicina, Universidade de São Paulo, São Paulo, Brazil; V MD, PhD. Attending Physician, Department of Internal Medicine, Clinical Immunology and Allergy Service, Hospital das Clínicas, Faculdade de Medicina, Universidade de São Paulo, São Paulo, Brazil; VI MD, PhD. Clinical Pathologist and Director of Clinical Biochemistry Service, Central Laboratory Division, Hospital das Clínicas, Faculdade de Medicina, Universidade de São Paulo, São Paulo, Brazil; VII MD, PhD. Full Professor, Department of Pathology, Faculdade de Medicina, Universidade de São Paulo, and Director of the Central Laboratory Division, Hospital das Clínicas, Faculdade de Medicina, Universidade de São Paulo, São Paulo, Brazil

**Keywords:** Clinical laboratory techniques, Blood chemical analysis, Calcium, Practice management, medical, Decision making, Técnicas de laboratório clínico, Análise química do sangue, Cálcio, Administração da prática médica, Tomada de decisões

## Abstract

**CONTEXT AND OBJECTIVE:**

This study was motivated by the recent excessive increase in requests for blood calcium determinations and laboratory tests in general, in the Hospital das Clínicas complex of Faculdade de Medicina, Universidade de São Paulo (HCFMUSP). Its aim was to suggest rules for the determination of total and ionized calcium in our intensive care units, emergency department, wards and outpatient services, thus contributing towards improving the quality of medical care and achieving more appropriate use of human and financial resources.

**DESIGN AND SETTING:**

Critical analysis on clinical and laboratory data and the pertinent scientific literature, conducted by the study group for rational clinical laboratory use, which is part of the Central Laboratory Division, HCFMUSP.

**METHODS:**

The study group reviewed scientific publications, statistics and clinical and laboratory data concerning requests for total and ionized calcium determinations in the settings of intensive care units, emergency department, wards and outpatient services.

**RESULTS:**

From this critical analysis, clinical decision flow diagrams aimed at providing guidance for ordering these tests were constructed.

**CONCLUSIONS:**

Use of the proposed flow diagrams may help to limit the numbers of inappropriate requests for ionized and total calcium determinations, with consequent reductions in the number of tests, risks to patients and unnecessary costs.

## Abstract (pt)

**CONTEXTO E OBJETIVO:**

Este trabalho foi motivado pelo recente aumento excessivo de solicitações de dosagem de cálcio no sangue, assim como de exames laboratoriais em geral, no complexo do Hospital das Clínicas da Faculdade de Medicina da Universidade de São Paulo (HCFMUSP). Seu objetivo foi sugerir regras para a determinação de cálcio total e iônico nas nossas unidades de terapia intensiva, pronto-socorro, enfermarias e ambulatórios e contribuir para a melhoria da qualidade da assistência médica, com utilização mais adequada dos recursos humanos e financeiros.

**TIPO DO ESTUDO E LOCAL:**

Análise crítica de dados clínicos, laboratoriais e da literatura médica pertinente, realizada pelo grupo de estudos para o uso racional do laboratório clínico, vinculado à Divisão de Laboratório Central do HCFMUSP.

**MÉTODOS:**

O grupo de estudos reviu publicações científicas, estatísticas e dados clínico-laboratoriais relativos às solicitações de cálcio total e iônico nos ambientes das unidades de terapia intensiva, prontos-socorros, enfermarias e ambulatórios.

**RESULTADOS:**

A partir dessa análise crítica, foram construídos fluxogramas de decisão clínica que visam orientar a requisição desses testes.

**CONCLUSÕES:**

A utilização dos fluxogramas propostos pode ajudar a limitar a solicitação inadequada das dosagens de cálcio total e iônico, com consequente redução do número de exames, de riscos para os pacientes e de custos desnecessários.

## INTRODUCTION

Over recent years, there has been a growing trend favoring extensive use of technology in medical practice, strongly supported by both the lay and the scientific media, with the intention of benefiting patients and society.^1^ As a side effect, excessive and inappropriate use of laboratory tests is leading to neglect of the logical primacy of clinical reasoning in diagnostic investigations and in follow-up,^2^ thereby increasing the chance of cognitive errors.^3^ Moreover, frequent collection of blood and other biological materials for laboratory tests may lead to significant blood losses,^4^ thus also increasing the risk of infections^5^ and false positive test results. In addition, this increases costs^6^ and overburdens nursing and laboratory teams.

The growing demand for laboratory tests can be ascribed to several factors, such as:

- The worldwide increase in longevity and associated comorbidities;^7^ ^,^ ^8^
- Overvaluation of laboratory tests at the expense of history-taking and physical examination;^3^
- Influence of technology and of the lay and scientific media;^1^ ^,^ ^9^
- Ignorance of costs;^7^
- Defensive medicine;^10^
- High complexity cases;^7^
- Insecurity or inexperience;^7^
- Lack of institutional guidelines, thus allowing arbitrary choice of laboratory tests.^6^

For all of the above reasons, there has been a steady increase in test ordering and in the related costs at our hospital, like in other institutions worldwide. This can be exemplified by blood calcium determinations. It should be stressed that in our institution, there are no formal restrictions in relation to ordering of any kind of test.

In 2012, the Central Laboratory Division of Hospital das Clínicas, Faculdade de Medicina, Universidade de São Paulo (HCFMUSP) recorded 233,955 total calcium determinations, which were performed at a direct cost of US$ 38,801.00 (R$ 79,544.70). On the other hand, over the same period, our records show 297,539 ionized calcium determinations at a direct cost of US$ 404,767.00 (R$ 829,773.89). These data are worrisome from a medical and financial standpoint and raise some serious questions:

- Were all these ionized calcium determinations really necessary?
- Is there actually a need to measure both ionized and total calcium?
- Are there any situations in which it is advantageous to measure ionized rather than total calcium?
- When are total calcium determinations sufficient to make medical decisions?
- Are there any situations in which ionized calcium determination must be avoided?
- Are there any situations in which calcium determinations must be repeated before 24 hours have elapsed?

## OBJECTIVE

The aim of this study was to provide suggested rules for total and ionized calcium determinations in our intensive care units, emergency department, wards and outpatient services thus contributing towards improving the quality of medical care and achieving more appropriate use of human and financial resources. They were formulated by our study group for rational laboratory test use, which is linked to the Central Laboratory Division of HCFMUSP. This group has been working to establish guidelines for rational ordering of costly or highly requested tests in our institution, since 2010.

## METHODS

HCFMUSP is a university hospital providing high-complexity care in São Paulo, Brazil, with 933 beds, of which 142 are in intensive care units. Our monthly average in 2012 was around 15,000 consultations for the emergency department and 92,000 for the outpatient services.

Our study group was created to promote rational use of the clinical laboratory. It is formed by seven physicians belonging to the staff of the hospital, and all of them have worked in the fields of intensive care, internal medicine, outpatient care, emergency medicine, clinical pathology or hospital administration for at least 15 years.

In order to draw up guidance algorithms for ordering ionized and total blood calcium determinations, this group attended weekly meetings, over a six-month period, in order to discuss and review theoretical matters and to study the data registers held by the Central Laboratory Division. The orders registered were examined with regard to the following: frequency; direct and indirect costs; blood collection; storage and processing of biological material; analytical methods and interferences with their precision and accuracy; interpretation of results within different scenarios such as intensive care units, emergency departments, wards and outpatient settings; and the reported medical management. Whenever necessary, specialists were invited to clarify specific questions regarding, for example, collection routines and analytical techniques, or calcium metabolism issues. 

Decision algorithms were chosen, on the assumption that they provided the most practical form for routine usage. These algorithms were approved by the Executive and Clinical Boards of Directors of the hospital, to be conveyed to clinical staff, residents, trainees and interns through electronic media, leaflets, posters and banners strategically located throughout the hospital. 

## RESULTS

We noticed that the number of ionized calcium determinations in our intensive care units was three times greater than the number of total calcium determinations. However, ionized calcium determinations in these units only accounted for 20% of the total for the institution. 

At the other extreme, in outpatient services, ionized calcium determinations represented 45% of the calcium measurements, i.e. almost the same quantity as for total calcium. 

**Figure 1:**
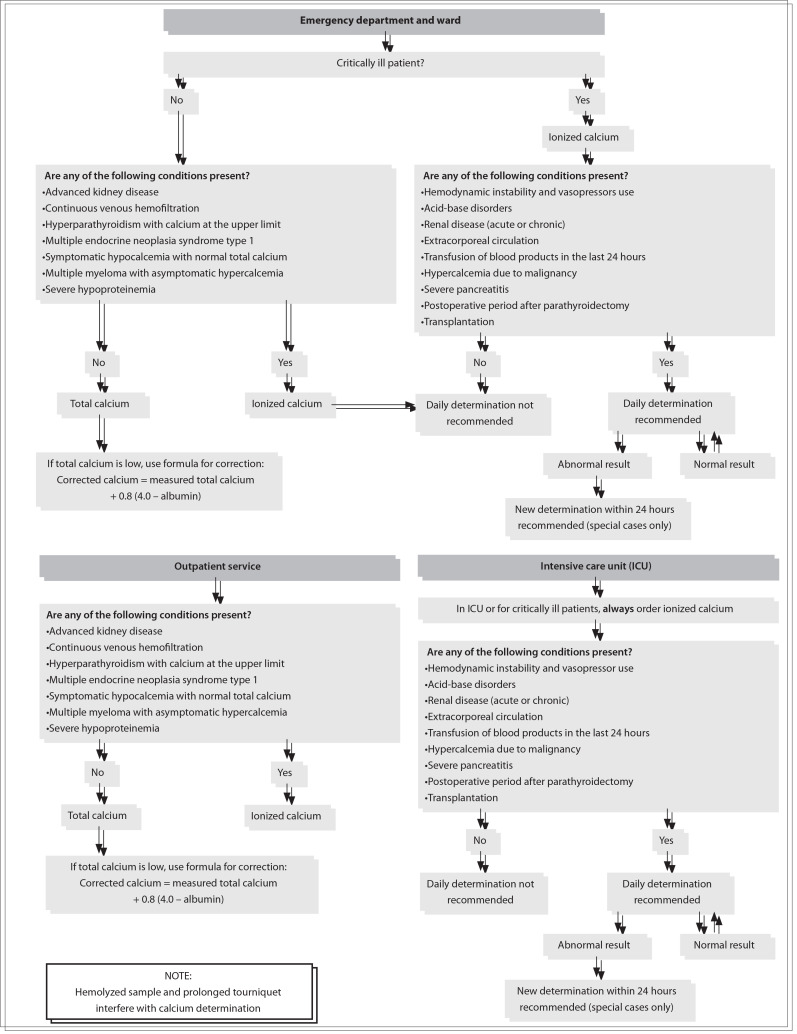
Recommendations for total and ionized calcium determination.

Figure 1 summarizes our recommendations for total and ionized calcium determinations, to be communicated to our entire clinical staff. Taking into account the above data, and in accordance with the algorithm shown in Figure 1, which was constructed through our group's expertise, the most important point in defining the choice between ionized and total calcium determination is the severity of the clinical condition. 

For critical patients, ionized calcium determinations must always be selected. They can be repeated every 24 hours or after shorter intervals, depending on the test results and on the need to check the impact of therapy. This recommendation is justifiable because critical patients usually present metabolic, hemodynamic and acid-base disorders that either lead to erroneous interpretations from total calcium determinations or give rise to acute changes in total calcium values that require correction (for example: hypoalbuminemia, metabolic acidosis and alkalosis, hyperventilation etc.). In the case of metabolic, endocrine or renal diseases (including those requiring hemodialysis), which can lead to acute changes in blood calcium levels, ionized calcium determinations are still preferred, though daily determinations are seldom justifiable. This applies to non-critical, stable, ward and emergency department inpatients, as well as to outpatients. 

In every other situation, especially for outpatients and ward patients undergoing follow-up or for diagnostic investigation of conditions that usually lead only to slow or occasional calcium blood changes, total calcium determination is preferable, when necessary. Low calcium values may need correction by means of the McLean-Hastings equation (see Discussion). 

Simultaneous determination of ionized and total calcium is never justifiable, since both tests measure the same parameter, i.e. the calcium level. 

## DISCUSSION

Both total and ionized calcium can be determined in blood. A colorimetric method is used for determining total calcium in serum or heparinized plasma, while direct measurement of ionized calcium requires equipment coupled to an ion-specific electrode, which can detect the element in total blood, plasma or serum. 

Currently, most blood-gas analyzers are able to determine ionized calcium, concomitantly with other electrolytes. These methodological advances have made calcium determination much easier, but, as an undesirable side effect, orders for these tests from physicians have skyrocketed. 

**Chart 1:**
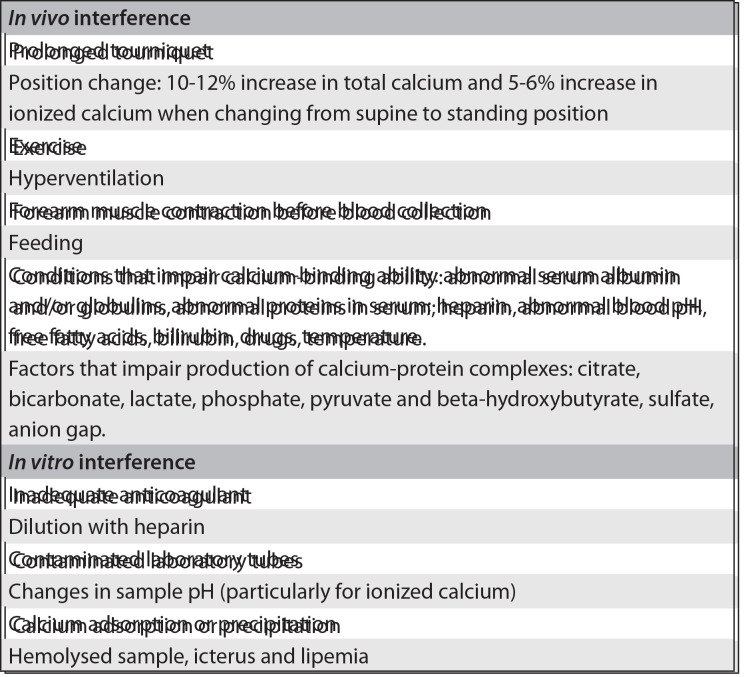
Pre-analytical factors that may interfere with the results from total and ionized calcium determinations14

In medical practice, the choice between total or ionized calcium measurement fundamentally depends upon the patient's clinical status and, secondarily, on technical issues. Some pre-analytical conditions^13^ (Chart 1)^14^ may exert an influence on the laboratory results for both total and ionized calcium and should be taken into account by the physician ordering the test. 

Ionized calcium levels in blood may vary more than total calcium levels, due to physiological factors, thus making ionized calcium the preferred test in cases of acute unstable clinical conditions, such as those occurring in patients presenting a critical health condition. The main factors that can induce significant changes to the blood level of the ionized fraction are detailed below and should be taken into account in the clinical-laboratory correlation:^15^ ^-^ ^18^

- Physical activity: Moderate physical exercise may elevate ionized calcium levels. This effect is related to lower pH and bicarbonate levels and higher lactate, albumin and total calcium occurring during physical activities.
- Body position: Changes in body position can affect blood protein levels, concentrations of protein-bound molecules and concentrations of low molecular weight ions. These changes occur due to leakage of fluid from blood vessels, and to increases in both muscle tonus and hydrostatic pressure.
- Feeding: After meals, a temporary decrease in ionized calcium levels of about 5.4% may occur. Several factors may contribute towards this phenomenon, such as: a) higher blood pH; b) an increase in blood protein concentration; or c) an increase in blood bicarbonate and phosphate concentration. All of these factors may act to increase the formation of calcium complexes with albumin and other ions.
- Ventilation: Respiratory alkalosis may decrease the ionized calcium concentration by 0.2 mg/dl or 0.05 mmol/l for each 0.1 unit of increase in pH.
- Circadian variation: Ionized calcium may vary by around 4 to 10% over the course of the day. This is possibly due to feeding, changes in acid-base balance and sleeping.
- Effects of the pH: Binding of calcium to proteins and anions is influenced by the pH *in vitro* and *in vivo*. Albumin is the main calcium-binding protein. An elevation of pH in a blood sample enhances the binding of calcium to albumin, with a consequent fall in the levels of ionized calcium. Conversely, a lower pH may increase the levels of ionized calcium. It has been estimated that there is a change of about 5% in ionized calcium for each 0.1 unit of change in pH. Because of the inverse relationship between pH and ionized calcium levels, the analysis must be done at the same pH as that of the patient. Therefore, the sample must either be analyzed immediately after it has been drawn or be preserved in order to avoid changes in pH.
- Requirements of the sample: Analyses on ionized calcium can be done on total blood with heparin, heparinized plasma or serum. The sample can be sent to the laboratory in a syringe or in a laboratory tube and must be kept sealed and only be opened at the time of the analysis, in order to avoid loss of CO_2_, which could result in elevation of the pH. Samples must be processed as soon as possible, in order to minimize the effects of the glycolysis induced by anaerobic metabolism of red and white blood cells.
- Sample stability: Processing of total blood samples for the analysis of ionized calcium, like that of blood gases, should be undertaken in up to 30 minutes. Samples collected in laboratory tubes and centrifuged to obtain serum or plasma remain stable for several hours if kept at 25 °C and for several days if kept at 4 °C, providing that they remain sealed.

Changes to the blood levels of serum proteins, particularly albumin, may result in changes to the total serum calcium content, with no effect on ionized calcium. However, variations in blood pH may change the concentration of ionized calcium.^19^

In view of the many factors that can artificially influence calcium blood levels, formulae have been developed for correcting laboratory calcium determinations. McLean and Hastings proposed the most widely used formula, which is shown below, for correction of the total calcium values when the plasma protein levels are altered:^15^ ^,^ ^20^

Corrected total calcium (mg/dl) = total calcium (mg/dl) + 0.8 x (4.0 - albumin (g/dl))

Following the same model, many formulae were proposed in the literature, with the aim of estimating the concentration of ionized calcium. These formulae include several factors, such as the blood levels of total protein and/or albumin, bicarbonate levels, globulin levels and the anion gap. However, the results obtained through these formulae do not always correlate with the values obtained from direct determination of ionized calcium, and therefore they should be abandoned.^21^

At HCFMUSP, no more than 20% of the requests for laboratory determinations of ionized calcium were made in relation to patients who were being treated in intensive care units. This means that 80% of the requests were made in relation to patients whose disease severity did not always justify ordering ionized calcium tests in preference to total calcium tests. For example, the numbers of requests for ionized calcium and total calcium tests for outpatients were approximately equal. The same reasoning as above, i.e. that disease severity is too low to justify requests for ionized calcium determination in this situation, applies here. 

Among the outpatients, requests for ionized calcium determinations accounted for 45% of all requests for ionized calcium tests from the whole hospital complex (Table 1). Moreover, for many patients, both tests were requested simultaneously, even though this was not warranted in most situations.

**Table 1:** Orders for total and ionized calcium determinations in 2012

	Total calcium	Ionized calcium
ICU	15,595	46,671
Emergency Department	27,539	20,674
Wards	38,090	62,489
Outpatient Services	101,222	103,937
Others*	51,509	63,768
Total	233,955	297,539

* Includes re-analysis on altered results

ICU = intensive care unit.

## CONCLUSIONS

If the severity and priority criteria summarized and illustrated in Figure 1 are followed, requests for determining ionized and total calcium can be made in a more orderly fashion, with possible consequent decreases in the number of tests ordered, risks to patients and unnecessary costs.
